
JOURNAL INFORMATION
==============================
NLM Title Abbreviation: Wirtschaftsdienst
Journal ID: Wirtschaftsdienst
NLM Journal ID: 101086612

Wirtschaftsdienst (Hamburg, Germany : 1949)

ISSN: 0043-6275

ARTICLE INFORMATION
==============================
PMCID: PMC7584852
PMID: 33132455
DOI: 10.1007/s10273-020-2769-1
Article ID: 2769
Article version: 1
Subjects: Ökonomische Trends

Muster der Erwerbslosigkeit in der COVID-19-Krise

Patterns of unemployment in the COVID 19 crisis

Wolf André wolf@hwwi.org

grid.435054.3 0000 0001 2227 8589 Hamburgisches WeltWirtschaftsInstitut (HWWI), Oberhafenstraße 1, 20097 Hamburg, Deutschland
Electronic publication date: 2020 Oct 24
Print publication date: 2020
Volume: 100
Issue: 10
First page: 815
Last page: 816
Copyright: © Der/die Autor(en) 2020
License: Dieser Artikel wird unter der Creative Commons Namensnennung 4.0 International Lizenz (https://creativecommons.org/licenses/by/4.0/deed.de) veröffentlicht.
Open Access wird durch die ZBW — Leibniz-Informationszentrum Wirtschaft gefördert.
License URL: https://creativecommons.org/licenses/by/4.0/

issue-copyright-statement: © The Author(s) 2020

==============================

Die globale COVID-19-Pandemie und die damit verknüpften krisenpolitischen Interventionen haben einen in dieser Form bislang einmaligen kombinierten Angebots- und Nachfrageschock auf den Weltmärkten ausgelöst. Demgegenüber fallen die Auswirkungen auf den deutschen Arbeitsmarkt, auch dank der Fördermaßnahmen im Bereich der Kurzarbeit, bislang eher moderat aus. Im Juli 2020 lag die Arbeitslosenquote deutschlandweit bei 6,3 %, und damit 1,2 Prozentpunkte höher als im Juli 2019 (5,1 %). Ein solcher Anstieg ist grundsätzlich für alle Personengruppen zu beobachten, allerdings in unterschiedlichem Ausmaß (vgl. Abbildung 1). So stieg im Vorjahresvergleich die Arbeitslosenquote der Männer (von 5,3 % auf 6,7 %) etwas stärker an als die der Frauen (von 4,9 % auf 5,9 %). Auch fiel bei älteren Erwerbspersonen über 55 Jahre dieser Anstieg (von 5,2 % auf 6,1 %) vergleichsweise schwach aus. Zugleich sind Ausländer (Anstieg von 12,1 % auf 15,5 %) gegenüber Inländern (von 4,1 % auf 5,1 %) wesentlich stärker betroffen.

Es liegt nahe, hierin einen Zusammenhang zu der spezifischen Natur des COVID-Schocks zu sehen. Dessen unmittelbare Wirkung war zunächst eine durch die Beschränkungen ausgelöste Angebotsrestriktion, und zwar in stark asymmetrischer Form, denn Zwangsschließungen wurden nur für bestimmte Bereiche des Handels und der personenbezogenen Dienstleistungen verordnet. Auch die sekundären Nachfrageschocks haben sich nicht in gleicher Weise auf die verschiedenen Wirtschaftszweige ausgewirkt, da sie neben der grundsätzlichen Konsumneigung auch die Konsummuster beeinflusst haben. Dies hat erwartungsgemäß zu einer stark unterschiedlichen Betroffenheit von Berufsgruppen durch die Krise geführt.

Betrachtet man die fünf Berufsgruppen (gemäß Klassifikation der Berufe, KldB 2010, 3-Steller-Ebene) mit der im Vergleich von Juli 2019 zum Juli 2020 stärksten absoluten Zunahme im bundesweiten Bestand an Arbeitslosen,[1] so sind dies „Lagerwirtschaft, Post, Zustellung, Güterumschlag“ (+ 59.875), „Verkauf (ohne Produktspezialisierung)“ (+ 41.872), „Reinigung“ (+ 41.218), „Speisenzubereitung“ (+ 33.579) und „Büro und Sekretariat“ (+ 32.632) (vgl. Abbildung 2). Eine für die zukünftige Entwicklung relevante Frage ist, inwieweit die beobachteten Unterschie-

1 Zuordnung gemäß Zielberufen.

Abbildung 1
**Arbeitslosenquoten nach Personengruppen in Deutschland**

Abbildung 2
**Arbeitslose nach Zielberufen in Deutschland**
Bestände in 1.000

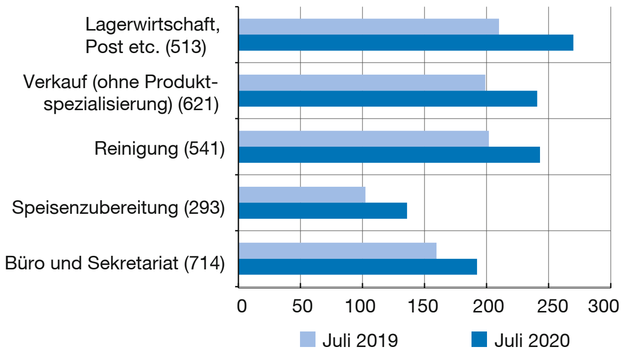

In Klammern: Nummer der Systematik der Klassifikation der Berufe, KldB 2010.

de zwischen soziodemografischen Gruppen durch ihre unterschiedliche Verteilung auf diese besonders betroffenen Berufsgruppen zu erklären sind. Deskriptive Anhaltspunkte sind dafür im Datenbestand zu finden.

In allen fünf Berufsgruppen mit Ausnahme von Lagerwirtschaft (513) waren Frauen zum 30.6.2019 im Vergleich zu ihrem Beschäftigtenanteil insgesamt (46,2 %) deutlich überproportional vertreten. In den Gruppen „Büro und Sekretariat" und „Reinigung" betrug ihr Anteil sogar 79,3 % bzw. 74,2 % (Statistik der Bundesagentur für Arbeit, 2019). Zugleich ist allerdings im Jahresvergleich Juli 2019 bis Juli 2020 in einer Mehrheit dieser Gruppen der Bestand an Arbeitslosen unter den Männern prozentual deutlich stärker gestiegen als unter den Frauen. Im besonderen Maße gilt dies für die Gruppen „Reinigung" und „Speisenzubereitung". Im Bereich „Speisenzubereitung" stieg der Bestand an männlichen Arbeitslosen um 51,4 %, an weiblichen Arbeitslosen aber nur um 20,9 %. Im Bereich „Reinigung" beträgt die Zunahme bei den Männern 32,3 %, bei den Frauen 17,6 %. Während Frauen im Hinblick auf ihre Berufswahl strukturell stärker von der Krise betroffen sind, haben sie innerhalb der besonders betroffenen Berufsgruppen mehrheitlich einen relativ schwächeren Netto-Zugang in die Arbeitslosigkeit erlebt als ihre männlichen Berufskollegen. Als Ursache kämen geschlechtsspezifische Unterschiede in der Verteilung auf Tätigkeitsbereiche innerhalb dieser Gruppen (d. h. einer tieferen Gliederungsebene) in Betracht. Ein Zusammenhang könnte auch zu den berufsbezogenen Anforderungsniveaus, d. h. der vertikalen Dimension der Berufeverteilung, bestehen. Auch hier zeigen sich Unterschiede: So ist in den Gruppen „Reinigung" und „Speisenzubereitung" die Zahl an arbeitslos gemeldeten Helfern im Betrachtungszeitraum deutlich stärker gestiegen als unter den Fachkräften. Unter den Helfern sind allerdings jeweils die Frauen überproportional vertreten, sodass dies nicht die stärkere Inzidenz bei den Männern erklären kann.

Einen andersgearteten Aussagegehalt besitzt die Berufeverteilung für die Unterschiede nach Altersgruppen. Die insgesamt weniger stark von Arbeitslosigkeit betroffene Alterskohorte 55+ war zum 30.6.2019 unter den betrachteten fünf Berufsgruppen im Schnitt leicht überdurchschnittlich vertreten. Mit Ausnahme von „Büro und Sekretariat" stellt sie zugleich in allen betrachteten Berufen die Kohorte mit dem geringsten Zuwachs an Arbeitslosigkeit im Jahresvergleich dar. In diesem Fall bestehen Hinweise auf einen Zusammenhang zu den Anforderungsniveaus: Der Anteil Helfer ist erwartungsgemäß unter den älteren Arbeitslosen in allen betrachteten Gruppen kleiner als unter den Jüngeren, ihre Betroffenheit durch eine spezifisch Ungelernte bedrohende Arbeitslosigkeit damit geringer.

Im Hinblick auf die Verteilung nach Nationalität stechen die Berufsgruppen „Reinigung", „Speisenzubereitung" und „Lagerwirtschaft" mit einem deutlich überdurchschnittlichen Ausländeranteil hervor. Damit scheint der überproportionale Anstieg der Arbeitslosigkeit unter Ausländern also durchaus in Zusammenhang mit der Berufswahl zu stehen. Zugleich haben aber auch innerhalb dieser Gruppen Ausländer einen jeweils stärkeren Zuwachs an Arbeitslosigkeit verzeichnet als Inländer. Dieser könnte sich zum Teil ebenfalls über Unterschiede im Anforderungsniveau erklären: der Anteil an Fachkräften fällt in all diesen Berufsgruppen unter ausländischen Arbeitslosen geringer aus als unter Inländern. Eine sich vor allem in der Entlassung Geringqualifizierter äußernde Krise trägt damit in besonderem Maße zu Arbeitslosigkeit unter Ausländern bei. Somit ergibt sich, dass ausländische Erwerbstätige gleich in doppeltem Maße negativ vom Corona-Schock betroffen sind (Statistik der Bundesagentur für Arbeit, 2020c).

Diese vorläufigen deskriptiven Ergebnisse verdeutlichen, dass die Frage des Tätigkeitsfeldes sowohl in horizontaler als auch in vertikaler Dimension eine wichtige Rolle bei der Verteilung der ökonomischen Wohlfahrtsschäden aus der Pandemie in der Gesellschaft spielen kann. Damit verknüpft ist die Frage, inwieweit die Arbeitsnachfrage in den besonders betroffenen Berufsgruppen auch längerfristig durch hiermit verbundene Struktureffekte beeinträchtigt bleiben wird. Dies wird unter anderem davon abhängen, ob nach dem Auslaufen der gegenwärtig bestehenden Kurzarbeiterförderung entsprechende Nachfolgeregelungen greifen werden, aber auch, ob ein nachhaltiger Umbau von Arbeitsplatzumgebungen mit Blick auf Mitarbeiter- und Kundenschutz gelingt. Um den Zusammenhang zwischen krisenbedingten Erwerbsverläufen und soziodemografischem Hintergrund näher ergründen zu können, bedarf es zukünftig der Auswertung detaillierter Mikrodaten. Die Makroebene liefert allerdings bereits deutliche Hinweise, dass Fragen der Qualifizierung und Weiterbildung mittelfristig stärkere gesellschaftspolitische Relevanz zukommen dürfte.

André Wolf
wolf@hwwi.org


Literatur

Statistik der Bundesagentur für Arbeit (2019), Beschäftige nach Berufen (KldB 2010), Juli.
Statistik der Bundesagentur für Arbeit (2020a), Arbeitslosigkeit im Zeitverlauf — Entwicklung der Arbeitslosenquote (Strukturmerkmale), August.
Statistik der Bundesagentur für Arbeit (2020b), Arbeitslose nach Zielberufen. Sonderauswertung, August.
Statistik der Bundesagentur für Arbeit (2020c), Arbeitslose nach Zielberufen und soziodemografischen Merkmalen. Sonderauswertung, August.
